# The association between NOTCH3 expression and the clinical outcome in the urothelial bladder cancer patients

**DOI:** 10.17305/bjbms.2021.6767

**Published:** 2022-01-24

**Authors:** Ana Ristić Petrović, Dragana Stokanović, Slavica Stojnev, Milena Potić Floranović, Miljan Krstić, Ivana Djordjević, Aleksandar Skakić, Ljubinka Janković Veličković

**Affiliations:** 1 Center for Pathology, University Clinical Center Niš, Niš, Serbia; 2 Department of Pathology, Faculty of Medicine, University of Niš, Niš, Serbia; 3 Department of Pharmacology, Faculty of Medicine, University of Niš, Niš, Serbia; 4 Scientific Research Center for Biomedicine, Faculty of Medicine, University of Niš, Niš, Serbia; 5 Clinic for Urology, University Clinical Center Niš, Niš, Serbia; 6 Department of Surgery, Faculty of Medicine, University of Niš, Niš, Serbia

**Keywords:** Urothelial bladder cancer, clinical outcome, NOTCH pathway, NOTCH3, immunohistochemistry

## Abstract

Disrupted NOTCH activity is a driving event in urothelial bladder cancer (UBC). After activation by hypoxia, the NOTCH3 receptor participates in tumor cell proliferation, acquisition of the epithelial-mesenchymal transition phenotype, and angiogenesis. The aim was to analyze the association of NOTCH3 expression with histopathological and clinical parameters and to determine its predictive impact on the clinical outcome in UBC patients. The present research included 614 UBC samples incorporated in paraffin tissue microarrays, evaluated by immunohistochemistry for NOTCH3 expression. The accrual period was 4 years, while the follow-up period was 2 years. The membranous expression was semi-quantified (0-3), and the mean degree was 1.81 ± 0.94. Criteria for semi-quantification, the NOTCH3 expression, were the intensity of the staining and the percentage of positive cells. The samples with negative (0) and weak (1) NOTCH3 immunohistochemical (IHC) score were considered negative, while the samples that showed moderate (2) and strong (3) expression were considered positive. Higher degree of positivity was associated with higher risk of cancer-specific mortality (*p* < 0.001). Independent predictors for cancer-specific mortality were NOTCH3 expression and high stage (*p* < 0.001). NOTCH3 expression was not a statistically significant predictor of recurrence-free survival (*p* = 0.816). This study indicated that NOTCH3 is a predictor of poor outcome, suggesting that the NOTCH3 could be potentially reliable IHC marker for selecting the UBC patients that would require more intensive follow-up, especially if they diagnosed in higher stage, with divergent differentiation in pathological report, and without recurrences which would lead them to more frequent medical assessments.

## INTRODUCTION

The NOTCH pathway plays an important role in tumor cells differentiation and further proliferation. Its involvement in tumorigenesis, tumor growth, and neovascularization is extremely complex and it is defined by tissue type and epithelial-mesenchymal transition (EMT) [1,2]. NOTCH signaling suppresses tumor growth and proliferation in several types of stratified epithelia [3]. This pathway controls tumor cells fate through interactions between neighboring cells, either leading the tumor cells in unstoppable proliferation or entirely opposite, leading them into apoptosis. Disrupted NOTCH activity is a driving event in urothelial bladder cancer (UBC) and NOTCH pathway mutations are equally common in superficial and invasive tumors [4]. In murine models, genetic inactivation of the NOTCH pathway accelerates bladder cancerogenesis, angiogenesis, and promotes the formation of highly invasive squamous cell carcinomas, with areas of mesenchymal features [5-7]. When it comes to NOTCH3 alterations, including amplification and upregulation, other research has already found that they are highly associated with tumor invasiveness and poor survival. After activation by hypoxia, the NOTCH3 receptor participates in tumor cell proliferation, acquisition of the EMT phenotype, and angiogenesis. Targeting NOTCH3 inhibits the growth of ovarian cancer and induces apoptosis [8]. It has been demonstrated that missense mutations in all NOTCH receptors resulted in loss of expression and aggressive behavior of the UBC [9,10]. However, not enough is known about the role of the NOTCH signaling pathway in UBC, especially regarding the importance of the NOTCH3 receptor.

The aim of this research was to evaluate the immunohistochemical (IHC) expression of NOTCH3 in UBC and to analyze the association of NOTCH3 expression with histopathological and clinical parameters, as well as, to determine its predictive impact on the clinical outcome: Cancer-specific, overall, and recurrence-free patients’ survival.

## MATERIALS AND METHODS

The current study included 614 samples from the primary lesion of UBC patients with pathologic stage pTa to pT4, who had undergone transurethral resection (TUR), partial resection, or radical cystectomy. All cases were diagnosed at the Center for Pathology, University Clinical Center Niš, Serbia, between March 2006 and December 2010. The study was approved by local ethical committee (12-15637-2/6). The patient’s average age was 66.38 ± 10.02 years, with approximately 3 times more male 469 (76.4%) patients. Hematuria was the first sign in 519 patients. The patients were diagnosed in pTa (31.1%), pT1 (45.9%), pT2 (17.3%), pT3 (3.9%), and pT4 (1.8%) stage. About 12% of the cases were from the settlements linked to the Balkan endemic nephropathy. In areas where BEN is endemic, the incidence of the urothelial cancer is significantly higher than in non-endemic regions [11]. Occupational exposure to known carcinogens (aromatic amines, nitrosamines, and polycyclic aromatic hydrocarbons) was observed in 47 UBC patients. Only several patients were with positive family history for the UBC (2.9%).

The accrual period was 4 years, while the follow-up period was 2 years. During that period, patients were monitored for recurrence and mortality.

The histological sections were processed from tissue fixed in 10% formalin by standard procedure and stained with hematoxylin and eosin (H&E). H&E-stained slides were used to assess histological grade (low and high grade), pathological stage (pT), growth of tumor (papillary/solid), the presence of carcinoma *in situ* (CIS), and divergent differentiation within the tumor (i.e., UBC with squamous differentiation and/or with glandular differentiation, and/or with trophoblastic differentiation), according to the WHO criteria [12]. This study included 73 (11.9%) cases of UBC with divergent differentiation, mostly squamous. Squamous differentiation was present in 53 UBCs, glandular in 12 cases, and trophoblastic differentiation in nine cases. Samples from 614 paraffin-embedded tissue blocks were extracted by 2 mm needle and incorporated in tissue microarrays. For constructing the microarrays, we chose the parts of the tumor with maximum of the angiogenic activity, according to microvessel density (established with CD34-positive endothelial cells) and high levels of vascular endothelial growth factor (VEGF) and VEGF receptor 1, which we established earlier and published previously [13-16]. Sections from created tissue microarrays were prepared overnight in a thermostat at a temperature of 58°C. After preparation, the slides were processed in a semi-automatic IHC diagnostic system (Ventana Inc.) and the IHC staining was performed using a rabbit polyclonal antibody to NOTCH3 (ab23426, Abcam, Cambridge, UK) at a concentration of 5 mg/ml. The slides were reviewed independently by three pathologists (ARP, SS, and LjJV). Interobserver discrepancies were resolved using a double-headed microscope. Since there is no validated scoring system for interpreting IHC staining for NOTCH3 and limited previous experience with this biomolecule in UBC, IHC cutoff value was defined according to scientific data that are available. We modified and semi-quantified previously published scoring systems for interpretation NOTCH3 expression [17-19] and other proteins expressed on the membrane [20,21]. Cytoplasmic or nuclear staining was considered non-specific and there were only few UBCs that appeared with aberrant NOTCH3 expression. Due to low number of negative samples, we semi-quantified NOTCH3 expression to assess whether the expression increases with higher tumor stage. Criteria for semi-quantification, the NOTCH3 expression, were the intensity of the IHC staining and the percentage of positive cells.

Intensity scoring was as follows: 0 – for no expression at all; 1 – weak intensity; 2 – moderate intensity; and 3 – strong intensity. In positive samples, the staining pattern on the membrane was uniform. The other criterion was the percentage of positive cells as follows: 0 – no positive cells, 1 – up to 20% of positive cells, 2 – >20-50% of positive cells, 3 – >50-80% of positive cells, and 4 – > 80% of positive cells. Considering both criteria, the final IHC score was as follows: 0-2 = negative (0); 3-4 = mild/weak (1); 5 = moderate (2); and 6-7 = strongly positive (3). The samples with negative (0) and weak (1) NOTCH3 IHC score were considered negative, while the samples that showed moderate (2) and strong (3) expression were considered positive.

### Ethical statement

The study was approved by local ethical committee (12-15637-2/6).

### Statistical analysis

All analyses were performed with the SPSS statistical package (SPSS v. 20.0, Chicago, USA). Data are presented as mean with standard deviation, for continuous variables, and as absolute numbers, for categorical variables. The association between various patients’ and UBCs’ characteristics, including NOTCH3 expression, was tested using parametric (Student’s t-test, ANOVA, Pearson’s test of correlation) or non-parametric tests (χ^2^-test). Cox regression modeling, univariate and multivariate, was performed to determine the predictive value of various independent variables for patients’ survival. The significance of NOTCH3 expression in survival prediction was depicted with Kaplan–Meier curves. *p* < 0.05 was considered statistically significant.

## RESULTS

**FIGURE 1:**
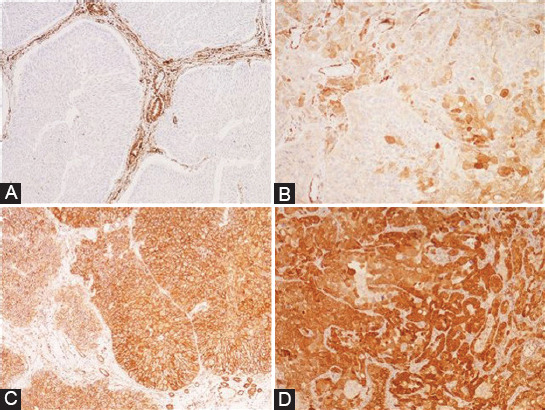
NOTCH3 expression in UBC; (A) NOTCH3-positive endothelial cells and NOTCH3-negative (0) tumor cells in pTa UBC (×100); (B) intermediate, focal NOTCH3 (1) staining in high-grade pT1 UBC (×200); (C) intermediate, diffuse NOTCH3 (2) staining in low-grade pT1 UBC (×100); (D) intense, diffuse NOTCH3 (3) staining in pT2 UBC (×200). UBC: Urothelial bladder cancer

IHC staining for NOTCH3 showed that the vast majority of the UBCs expressed NOTCH3 (91.5%), at certain degree. The intensity of the membranous expression was semi-quantified (0-3), and the mean degree was 1.81 ± 0.94 (Figure 1).

### NOTCH3 positivity and patients’ and UBC’s characteristics

Patients’ and UBC samples’ characteristics, according to NOTCH3 positivity, are shown in Table 1. Due to low number of negative samples, *post hoc* analysis using semi-quantified NOTCH3 staining was performed. The results obtained are presented in Table 2. There was a weak positive correlation between NOTCH3 positivity and patients’ age (*p* < 0.001). Furthermore, higher degree of NOTCH3 staining was observed in high-grade tumors (*p* < 0.001), staged pTa and pT1 (*p* < 0.001), and with no history of exposure to known carcinogens (*p* < 0.05).

**TABLE 1:**
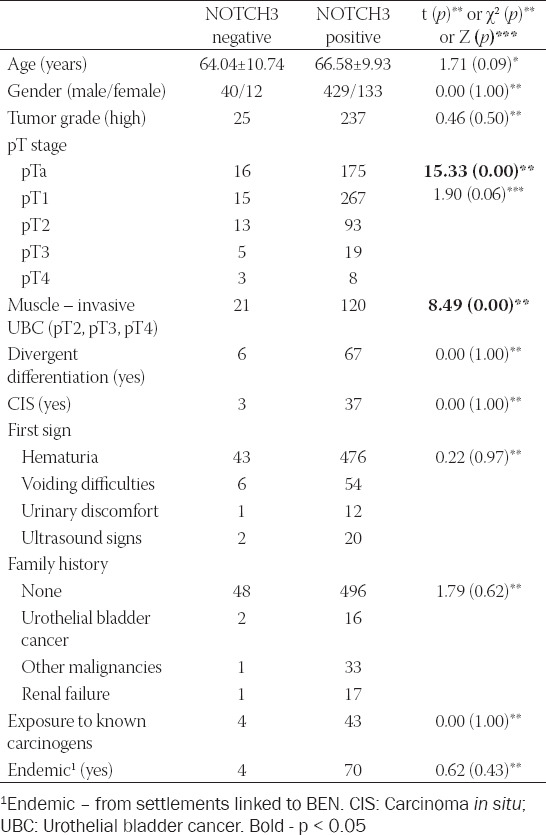
Histopathological and clinical characteristics of urothelial bladder cancer patients according to NOTCH3 expression

**TABLE 2:**
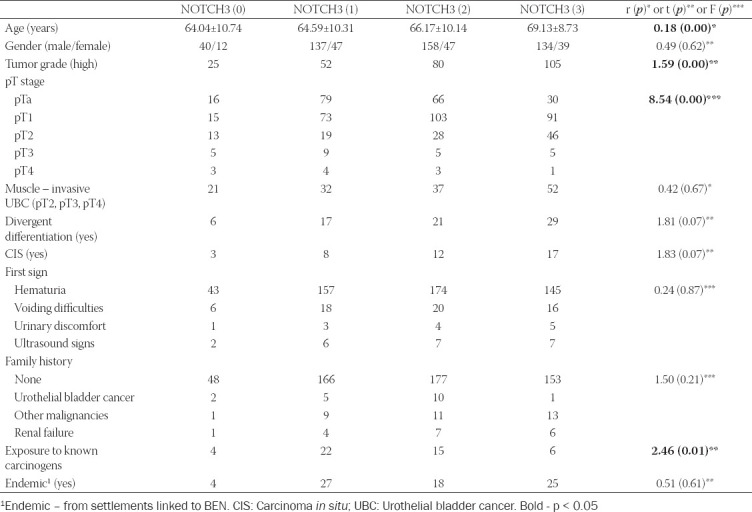
*Post hoc* analysis with semi-quantified NOTCH3 expression

### Overall and recurrence-free survival

The median follow-up in the study group was 45.0 (24.0-64.0) months. During this period, the mortality rate was 42.5%, UBC specific in most of the cases (69.7%). Recurrences occurred in 230 (37.5%) patients, mostly 1 time (58.7%). The median recurrence-free period was 12.0 (0.0-44.0) months. The differences in NOTCH3 expression according to survival are presented in Table 3. There was a weak negative correlation between NOTCH3 expression and the overall survival (ρ = −0.103, *p* < 0.001) and cancer-specific mortality (ρ = −0.068, *p* < 0.05).

**TABLE 3:**
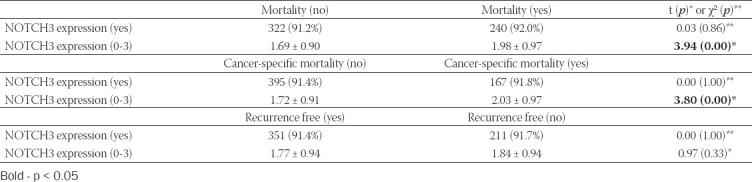
Patients’ survival according to NOTCH3 expression

**FIGURE 2:**
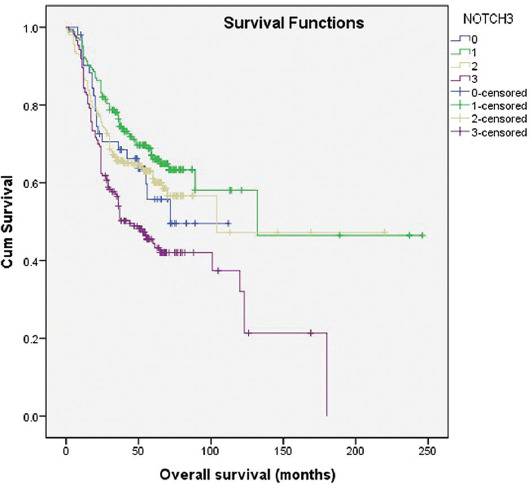
Kaplan–Meier survival curves showing overall survival of 614 bladder cancer patients with negative (0=no staining; 1=weak and/or focal staining) and positive (2=diffuse, intermediate staining; 3=strong, diffuse staining) NOTCH3 expression.

Figure 2 presents the overall survival of bladder cancer patients according to NOTCH3 expression (0-3). Each higher degree of positivity is associated with 1.3 times higher risk of mortality (*p* < 0.001).

Cox regression model adjusted for patients age, predicting overall mortality, was created (χ^2^=297.255, *p* < 0.001). Several predictors, identified by univariate modeling, were excluded from the multivariate model due to high colinearity. Four independent predictors were identified. The overall mortality increased 1.2 times with each higher degree of NOTCH3 expression (*p* < 0.01) and 2.1 times (*p* < 0.001) with each higher tumor stage. In contrast, each new recurrence increases survival 1.4 times (*p* < 0.001). Moreover, patients with divergent differentiation cancer had 1.5 times shorter survival (*p* < 0.05) (Table 4).

**TABLE 4:**
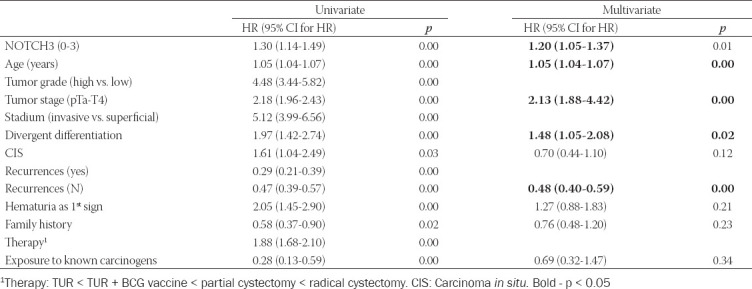
Cox regression model of overall survival

**FIGURE 3:**
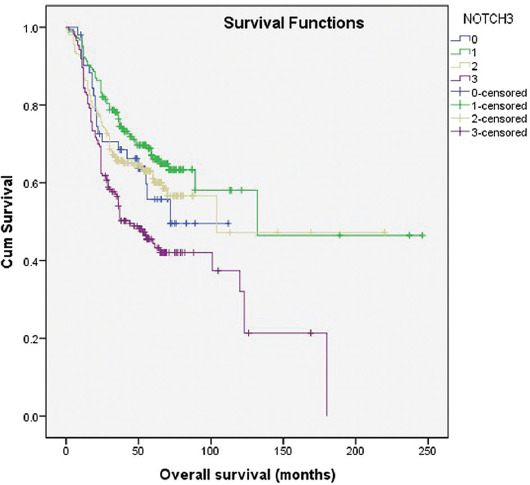
Kaplan–Meier survival curves showing cancer-specific survival of 614 bladder cancer patients with negative (0=no staining; 1=weak and/or focal staining) and positive (2= intermediate, diffuse staining; 3=strong, diffuse staining) NOTCH3 expression.

Concerning cancer-specific survival, each higher degree of positivity was associated with 1.4 higher risk of cancer-specific mortality (*p* < 0.001) (Figure 3).

Cox regression model, predicting cancer-specific ­mortality, was created (χ^2^=251.886, *p* < 0.001) and adjusted for patients age. NOTCH3 was shown to be an independent predictor of UBC-specific mortality – each higher degree of its expression increased the risk 1.3 times (*p* < 0.01). The other independent predictors of UBC-specific survival were pathological tumor stage (HR = 2.520, *p* < 0.001), divergent differentiation (HR = 1.631, *p* < 0.05), and the number of recurrences (HR = 0.515, *p* < 0.001) (Table 5). The same independent predictors were found for the overall mortality as well. After comparing the two models obtained, the predictive value of the number of relapses was higher for overall mortality, while NOTCH3 expression, tumor stage, and cancer divergent differentiation had greater value in predicting UBC-specific mortality.

**TABLE 5:**
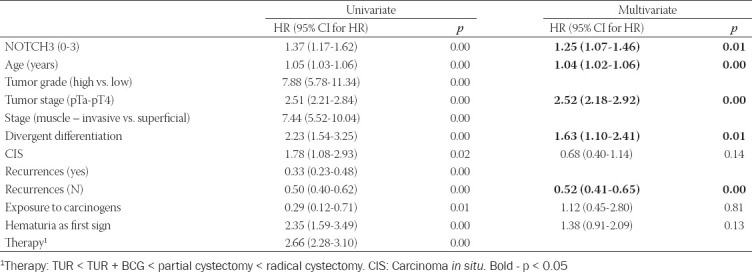
Cox regression model of UBC-specific survival

**FIGURE 4:**
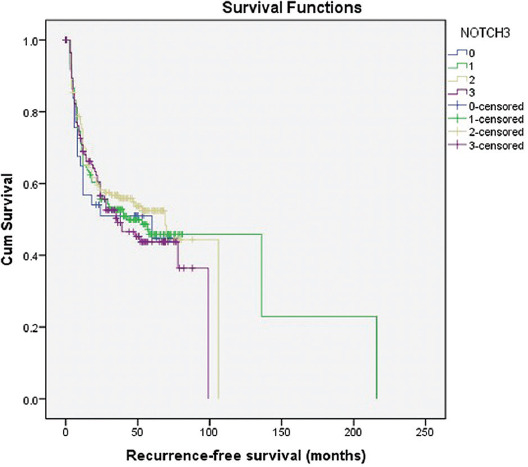
Kaplan–Meier survival curves showing recurrence-free survival of 614 bladder cancer patients with negative (0=no staining; 1=weak and/or focal staining) and positive (2=intermediate, diffuse staining; 3=strong, diffuse staining) NOTCH3 expression.

NOTCH3 expression was not a statistically significant predictor of recurrence-free survival (*p* = 0.816) (Figure 4).

## DISCUSSION

NOTCH signaling pathway mediates between hypoxia, EMT, and angiogenesis [22,23]. High levels of tumor hypoxia are associated with higher angiogenic activity and invasiveness through acquisition the EMT phenotype [24]. In a hypoxic environment, NOTCH signaling has been implicated in pericyte recruitment, microvessel sprouting, branching, and finally vessel maturation [25,26]. Considerable studies have shown that activated Notch pathway increases the possibility of metastasis with poor outcome on the basis of involvement in hypoxia, tumor perfusion, and angiogenesis [27,28]. Although Rampias et al. indicated the new tumor suppressor role for the NOTCH pathway, studies have revealed that not all NOTCH receptors have the same involvement in UBC [4]. NOTCH1 expression is decreased in UBC, suggesting a tumor-suppressive role. On the contrary, it has been demonstrated that NOTCH2 acts as an oncogene that promotes cell proliferation, aggressiveness, and metastasis through EMT, cell cycle progression, and maintenance of stemness [6]. NOTCH3 is activated by chronic hypoxia and it is widely expressed throughout tumor angiogenesis [29]. In ovarian cancer, NOTCH3 overexpression induces EMT, chemoresistance, and is associated with poor overall survival [30,31].

The present study showed that NOTCH3 expression correlates with UBC-specific mortality. Zhang et al. found that high NOTCH3 expression was associated with poor patient survival, which is in accordance with our results. Recent studies showed that NOTCH3 knockdown decreases UBC growth *in vivo*. Decreased NOTCH3 expression sensitizes urothelial cancer cells to cisplatin. Moreover, Zhang et al. suggested that decreasing the expression of NOTCH3 using histone deacetylase inhibitors is likely to become an effective therapeutic strategy for UBC [32].

NOTCH3 acetylation/deacetylation represents a key regulatory switch in the control of NOTCH signaling and might represent a suitable drug target for NOTCH3 sustained UBC. Targeting NOTCH3 is proposed as new promising therapy in other cancers as well [33-35].

Over 90% of our specimens showed a certain degree of NOTCH3 expression, suggesting that the NOTCH3 has relevant role in UBC. Up to date, the most reliable prognostic parameters in histopathological report are histological grade, presence of lymphovascular invasion, and concomitant CIS [36-38]. Regardless of the fact that the UBCs with divergent differentiation behave more aggressively, we did not find a statistical association between divergent differentiation and NOTCH3 expression. Nevertheless, the divergent differentiation had impact on the survival, both overall and UBC-specific survival. We evaluated the intensity of NOTCH3 expression in a semi-quantitative manner, which revealed that higher degree of NOTCH3 expression was observed in high-grade tumors and higher degree of positivity associated with higher risk of mortality. We identified NOTCH3 as an independent predictor of poor outcome. Our results indicate that NOTCH3 could be used as a marker of the UBC-specific mortality risk. Consistent with published data, we determined that NOTCH3 expression is associated with patients’ age, grade, and stage [39,40]. Better overall survival was found to be in association with recurrent disease and among patients treated with TUR followed with BCG vaccine instillation. In agreement with our results, Thiel et al. showed that the BCG treatment reduces the long-term risk of recurrence and progression in high-risk non-muscle invasive UBC patients [41].

Furthermore, our results showed that the number of recurrences during the follow-up period was a better predictor of cancer-specific survival compared to being recurrence free. As a potential survival predictor, we included total number of recurrences. We have found out that a higher number of recurrences lower the risk of death, each one by 40%. Even though it would be expected to observe more recurrences in patients with longer survival, we suppose that the recurrences were the reason for more frequent doctor-patient interaction and better overall treatment, leading to longer survival. Among all parameters analyzed, solely, the incidence of recurrences was associated with the increased risk of new occurrences. Similar findings were published recently [42]. Greater number of recurrences during the follow-up period was associated with lower tumor stage and positive family history. Unfortunately, in this study, NOTCH3 expression was not a statistically significant predictor of recurrence-free survival. Our results indicate that NOTCH3 expression could be a prognostic IHC marker for the UBC patients clinical follow-up, contributing to a more individual approach by selecting the patients who need to undergo control cystoscopy after a shorter time interval.

## CONCLUSION

This study indicated that the NOTCH3 is a predictor of poor outcome, suggesting that the NOTCH3 could be potentially reliable IHC marker for selecting the UBC patients that would require more intensive follow-up – especially if they are diagnosed in higher stage, with divergent differentiation in pathological report, and without recurrences which would lead them to more frequent medical assessments. Understanding the role of the NOTCH pathway, the interplay between NOTCH receptors, and their importance in the UBC could bring about new possibilities for better controlling one of the most prevalent cancers in urology.
